# Bis[4-(2-nitro­benzene­sulfonamido)­pyridinium] hexa­fluoro­silicate

**DOI:** 10.1107/S1600536808038191

**Published:** 2008-11-22

**Authors:** Ai-Rong Wang

**Affiliations:** a School of Chemistry and Chemical Engineering, Henan Institute of Science and Technology, Xinxiang 453003, People’s Republic of China

## Abstract

In the title compound, 2C_11_H_10_N_3_O_4_S^+^·SiF_6_^2−^, the short C—N distance [1.386 (2) Å] is indicative of a slight conjugation of N with the π electrons of the pyridinium ring, and with those of the sulfonyl group. The dihedral angle between the benzene and pyridinium rings is 77.48 (7)°. The crystal structure is stabilized by N—H⋯F and C—H⋯F hydrogen bonds. The Si atom of the anion lies on a special position.

## Related literature

For zwitterionic forms of *N*-aryl­benzene­sulfonamides, see: Li *et al.* (2007 ▶); Yu & Li (2007 ▶). For reference geometric data, see: Allen *et al.* (1987 ▶). Damiano *et al.* (2007 ▶) describe the use of pyridinium derivatives for the construction of supra­molecular architectures.
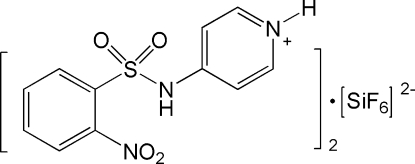


## Experimental

### 

#### Crystal data

2C_11_H_10_N_3_O_4_S^+^·SiF_6_^2−^
*M* *_r_* = 702.65
Monoclinic, 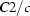

*a* = 22.691 (5) Å
*b* = 8.524 (2) Å
*c* = 14.776 (3) Å
β = 110.95 (3)°
*V* = 2669 (1) Å^3^
*Z* = 4
Mo *K*α radiation
μ = 0.35 mm^−1^
*T* = 113 (2) K
0.22 × 0.16 × 0.04 mm

#### Data collection

Rigaku Saturn CCD area-detector diffractometer
Absorption correction: multi-scan (*CrystalClear*; Rigaku/MSC, 2005 ▶) *T*_min_ = 0.927, *T*_max_ = 0.986
10620 measured reflections
3064 independent reflections
2456 reflections with *I* > 2σ(*I*)
*R*_int_ = 0.053

#### Refinement

*R*[*F* ^2^ > 2σ(*F* ^2^)] = 0.043
*wR*(*F* ^2^) = 0.111
*S* = 1.06
3064 reflections
212 parameters
H atoms treated by a mixture of independent and constrained refinement
Δρ_max_ = 0.38 e Å^−3^
Δρ_min_ = −0.49 e Å^−3^

### 

Data collection: *CrystalClear* (Rigaku/MSC, 2005 ▶); cell refinement: *CrystalClear*; data reduction: *CrystalClear*; program(s) used to solve structure: *SHELXS97* (Sheldrick, 2008 ▶); program(s) used to refine structure: *SHELXL97* (Sheldrick, 2008 ▶); molecular graphics: *SHELXTL* (Sheldrick, 2008 ▶); software used to prepare material for publication: *SHELXTL*.

## Supplementary Material

Crystal structure: contains datablocks global, I. DOI: 10.1107/S1600536808038191/lx2077sup1.cif

Structure factors: contains datablocks I. DOI: 10.1107/S1600536808038191/lx2077Isup2.hkl

Additional supplementary materials:  crystallographic information; 3D view; checkCIF report

## Figures and Tables

**Table 1:** Hydrogen-bond geometry (Å, °)

*D*—H⋯*A*	*D*—H	H⋯*A*	*D*⋯*A*	*D*—H⋯*A*
N1—H1N⋯F1^i^	0.86 (3)	1.96 (3)	2.789 (2)	162 (3)
N2—H2N⋯F1^ii^	0.73 (3)	1.97 (3)	2.690 (2)	171 (3)
C4—H4⋯F2^i^	0.95	2.35	3.141 (2)	141
C5—H5⋯F3^ii^	0.95	2.50	3.426 (3)	165

Symmetry codes: (i) 
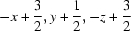
; (ii) 
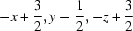
.

**Fig. 1.**
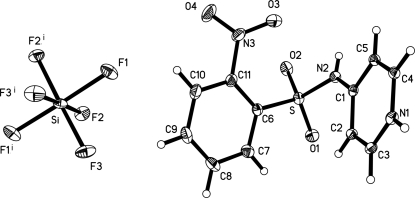
View of one molecule of the title compound showing the atom-labelling scheme. Displacement ellipsoids are drawn at the 35% probability level (arbitrary spheres for the H atoms). [Symmetry code: (i) -x + 2, y, -z + 3/2.]

## supplementary crystallographic information

### Comment

Organic pyridinium salts have been widely used in the construction of supramolecular architectures (Damiano *et al.*, 2007). As part of our ongoing studies of supramolecular chemistry involving the pyridinium rings (Li *et al.*, 2007), herein we report the crystal structure of the title compound, 4-(2-nitrophenylsulfonylamino)pyridinium hexafluorosilicate (Fig. 1).

In the cations of the title compound the short C—N distance [N2—C1 = 1.386 (2) Å] has a value between those of a typical C═N double and C—N single bond (1.47–1.50 Å and 1.34–1.38 Å, respectively; Allen *et al.*, 1987). This might be indicative of a slight conjugation of N with π electrons of the pyridinium ring, and with those of the sulfonyl group. The dihedral angle between the benzene ring and the pyridinium ring is 77.48 (7)°. The crystal structure is stabilized by N—H···F and C—H···F hydrogen bonds (Table 1).

### Experimental

A solution of 2-nitrobenzenesulfonyl chloride (2.2 g, 10 mmol) in CH_2_Cl_2_ (10 ml) was added dropwise to a suspension of 4-aminopyridine (0.9 g, 10 mmol) in CH_2_Cl_2_ (10 ml) at room temperature with stirring. The reaction mixture was stirred overnight. The yellow solid obtained was washed with warm water to obtain the title compound in a yield of 55.3%. A colorless single crystal suitable for X-ray analysis was obtained by slow evaporation of a fluorosilicic acid (10%) solution at room temperature over a period of a week.

### Refinement

The N-bound H atoms were located in a difference density Fourier map and freely refined. The C-bound H atoms were positioned geometrically (C—H = 0.95 Å) and refined as riding with *U*_iso_(H) = 1.2*U*_eq_(C).

### Crystal data

**Table:**

2C_11_H_10_N_3_O_4_S^+^·SiF_6_^2–^	*F*_000_ = 1432
*M**_r_* = 702.65	*D*_x_ = 1.749 Mg m^−^^3^
Monoclinic, *C*2/*c*	Mo *K*α radiation λ = 0.71073 Å
Hall symbol: -C 2yc	Cell parameters from 3772 reflections
*a* = 22.691 (5) Å	θ = 1.9–27.5º
*b* = 8.524 (2) Å	µ = 0.35 mm^−^^1^
*c* = 14.776 (3) Å	*T* = 113 (2) K
β = 110.95 (3)º	Block, colourless
*V* = 2669 (1) Å^3^	0.22 × 0.16 × 0.04 mm
*Z* = 4	

### Data collection

**Table:**

Rigaku Saturn CCD area-detector diffractometer	3064 independent reflections
Radiation source: rotating anode	2456 reflections with *I* > 2σ(*I*)
Monochromator: confocal	*R*_int_ = 0.053
Detector resolution: 7.31 pixels mm^-1^	θ_max_ = 27.5º
*T* = 113(2) K	θ_min_ = 1.9º
ω and φ scans	*h* = −22→29
Absorption correction: multi-scan(CrystalClear; Rigaku/MSC, 2005)	*k* = −10→11
*T*_min_ = 0.927, *T*_max_ = 0.986	*l* = −18→19
10620 measured reflections	

### Refinement

**Table:**

Refinement on *F*^2^	Secondary atom site location: difference Fourier map
Least-squares matrix: full	Hydrogen site location: difference Fourier map
*R*[*F*^2^ > 2σ(*F*^2^)] = 0.043	H atoms treated by a mixture of independent and constrained refinement
*wR*(*F*^2^) = 0.111	*w* = 1/[σ^2^(*F*_o_^2^) + (0.0513*P*)^2^ + 1.0092*P*] where *P* = (*F*_o_^2^ + 2*F*_c_^2^)/3
*S* = 1.06	(Δ/σ)_max_ < 0.001
3064 reflections	Δρ_max_ = 0.38 e Å^−^^3^
212 parameters	Δρ_min_ = −0.49 e Å^−^^3^
Primary atom site location: structure-invariant direct methods	Extinction correction: none

### Special details

**Table:**

Geometry. All esds (except the esd in the dihedral angle between two l.s. planes) are estimated using the full covariance matrix. The cell esds are taken into account individually in the estimation of esds in distances, angles and torsion angles; correlations between esds in cell parameters are only used when they are defined by crystal symmetry. An approximate (isotropic) treatment of cell esds is used for estimating esds involving l.s. planes.
Refinement. Refinement of F^2^ against ALL reflections. The weighted R-factor wR and goodness of fit S are based on F^2^, conventional R-factors R are based on F, with F set to zero for negative F^2^. The threshold expression of F^2^ > 2sigma(F^2^) is used only for calculating R-factors(gt) etc. and is not relevant to the choice of reflections for refinement. R-factors based on F^2^ are statistically about twice as large as those based on F, and R- factors based on ALL data will be even larger.

### Fractional atomic coordinates and isotropic or equivalent isotropic displacement parameters (Å^2^)

**Table:**

	*x*	*y*	*z*	*U*_iso_*/*U*_eq_	
S	0.61983 (2)	0.29097 (5)	0.52758 (4)	0.01969 (15)	
O1	0.58452 (8)	0.32136 (18)	0.42794 (11)	0.0273 (4)	
O2	0.63133 (8)	0.13308 (16)	0.56235 (12)	0.0282 (4)	
O3	0.69576 (9)	0.3188 (2)	0.75056 (13)	0.0473 (5)	
O4	0.78705 (10)	0.2230 (3)	0.7737 (2)	0.0843 (10)	
N1	0.56160 (8)	0.8430 (2)	0.63088 (13)	0.0192 (4)	
H1N	0.5583 (14)	0.942 (3)	0.639 (2)	0.052 (9)*	
N2	0.58416 (9)	0.3725 (2)	0.59405 (14)	0.0194 (4)	
H2N	0.5795 (14)	0.315 (3)	0.628 (2)	0.043 (9)*	
N3	0.74149 (10)	0.3008 (2)	0.72703 (15)	0.0363 (5)	
C1	0.57577 (9)	0.5317 (2)	0.60418 (14)	0.0167 (4)	
C2	0.57532 (9)	0.6398 (2)	0.53308 (14)	0.0177 (4)	
H2	0.5800	0.6058	0.4748	0.021*	
C3	0.56798 (9)	0.7955 (2)	0.54852 (15)	0.0193 (4)	
H3	0.5674	0.8704	0.5006	0.023*	
C4	0.56074 (9)	0.7410 (2)	0.70051 (14)	0.0195 (4)	
H4	0.5555	0.7784	0.7577	0.023*	
C5	0.56742 (9)	0.5837 (2)	0.68832 (14)	0.0185 (4)	
H5	0.5664	0.5109	0.7364	0.022*	
C6	0.69299 (10)	0.3905 (2)	0.55358 (15)	0.0192 (4)	
C7	0.70103 (11)	0.4743 (2)	0.47788 (16)	0.0258 (5)	
H7	0.6675	0.4795	0.4169	0.031*	
C8	0.75749 (12)	0.5503 (3)	0.49066 (18)	0.0309 (5)	
H8	0.7620	0.6078	0.4385	0.037*	
C9	0.80672 (11)	0.5435 (3)	0.57748 (19)	0.0307 (5)	
H9	0.8451	0.5965	0.5854	0.037*	
C10	0.80059 (10)	0.4591 (3)	0.65379 (18)	0.0295 (5)	
H10	0.8347	0.4530	0.7141	0.035*	
C11	0.74405 (10)	0.3839 (3)	0.64084 (16)	0.0249 (5)	
Si	1.0000	0.65155 (9)	0.7500	0.01731 (19)	
F1	0.94232 (7)	0.64561 (14)	0.79977 (10)	0.0303 (3)	
F2	0.96119 (6)	0.51233 (14)	0.67115 (9)	0.0265 (3)	
F3	0.96052 (7)	0.79039 (14)	0.67359 (9)	0.0333 (3)	

### Atomic displacement parameters (Å^2^)

**Table:**

	*U*^11^	*U*^22^	*U*^33^	*U*^12^	*U*^13^	*U*^23^
S	0.0207 (3)	0.0158 (3)	0.0229 (3)	−0.0010 (2)	0.0083 (2)	−0.00471 (19)
O1	0.0294 (9)	0.0304 (8)	0.0199 (8)	−0.0032 (7)	0.0060 (6)	−0.0071 (6)
O2	0.0324 (9)	0.0145 (7)	0.0426 (10)	0.0012 (7)	0.0196 (8)	−0.0020 (7)
O3	0.0360 (11)	0.0728 (14)	0.0333 (11)	0.0026 (10)	0.0127 (8)	0.0205 (10)
O4	0.0312 (11)	0.112 (2)	0.104 (2)	0.0222 (13)	0.0172 (12)	0.0855 (17)
N1	0.0217 (9)	0.0125 (8)	0.0228 (10)	0.0012 (7)	0.0073 (7)	−0.0012 (7)
N2	0.0221 (9)	0.0136 (8)	0.0242 (10)	0.0014 (7)	0.0104 (7)	0.0011 (7)
N3	0.0227 (10)	0.0443 (13)	0.0350 (12)	−0.0019 (9)	0.0019 (9)	0.0175 (10)
C1	0.0135 (9)	0.0149 (9)	0.0205 (10)	−0.0005 (8)	0.0045 (7)	−0.0011 (8)
C2	0.0195 (10)	0.0196 (10)	0.0138 (10)	0.0015 (8)	0.0060 (8)	−0.0017 (8)
C3	0.0184 (10)	0.0182 (10)	0.0195 (10)	0.0009 (8)	0.0048 (8)	0.0026 (8)
C4	0.0178 (10)	0.0244 (10)	0.0165 (10)	0.0018 (9)	0.0064 (8)	−0.0008 (8)
C5	0.0177 (10)	0.0195 (10)	0.0182 (10)	−0.0008 (8)	0.0063 (8)	0.0003 (8)
C6	0.0202 (10)	0.0168 (9)	0.0219 (11)	0.0020 (8)	0.0092 (8)	−0.0028 (8)
C7	0.0293 (12)	0.0270 (11)	0.0237 (12)	0.0011 (10)	0.0126 (9)	−0.0029 (9)
C8	0.0367 (14)	0.0297 (12)	0.0359 (14)	−0.0016 (11)	0.0247 (11)	−0.0002 (10)
C9	0.0225 (11)	0.0292 (11)	0.0457 (15)	−0.0018 (10)	0.0184 (10)	−0.0011 (11)
C10	0.0187 (11)	0.0289 (11)	0.0377 (14)	0.0019 (10)	0.0060 (9)	0.0040 (10)
C11	0.0216 (11)	0.0230 (10)	0.0295 (12)	0.0044 (9)	0.0083 (9)	0.0044 (9)
Si	0.0263 (4)	0.0132 (4)	0.0144 (4)	0.000	0.0098 (3)	0.000
F1	0.0431 (8)	0.0202 (6)	0.0393 (8)	0.0036 (6)	0.0292 (7)	0.0010 (5)
F2	0.0306 (7)	0.0222 (6)	0.0233 (7)	−0.0018 (6)	0.0056 (5)	−0.0054 (5)
F3	0.0505 (9)	0.0225 (7)	0.0248 (7)	0.0093 (6)	0.0110 (6)	0.0072 (5)

### Geometric parameters (Å, °)

**Table:**

S—O1	1.426 (2)	C4—H4	0.9500
S—O2	1.431 (2)	C5—H5	0.9500
S—N2	1.634 (2)	C6—C11	1.393 (3)
S—C6	1.779 (2)	C6—C7	1.393 (3)
O3—N3	1.216 (3)	C7—C8	1.387 (3)
O4—N3	1.214 (3)	C7—H7	0.9500
N1—C3	1.338 (3)	C8—C9	1.368 (3)
N1—C4	1.353 (3)	C8—H8	0.9500
N1—H1N	0.86 (3)	C9—C10	1.385 (3)
N2—C1	1.386 (2)	C9—H9	0.9500
N2—H2N	0.73 (3)	C10—C11	1.385 (3)
N3—C11	1.477 (3)	C10—H10	0.9500
C1—C2	1.395 (3)	Si—F3^i^	1.6597 (14)
C1—C5	1.395 (3)	Si—F3	1.6597 (14)
C2—C3	1.367 (3)	Si—F2	1.6756 (13)
C2—H2	0.9500	Si—F2^i^	1.6756 (13)
C3—H3	0.9500	Si—F1^i^	1.7161 (13)
C4—C5	1.368 (3)	Si—F1	1.7161 (13)
			
O1—S—O2	120.30 (10)	C7—C6—S	116.81 (16)
O1—S—N2	109.10 (10)	C8—C7—C6	120.7 (2)
O2—S—N2	104.47 (10)	C8—C7—H7	119.7
O1—S—C6	105.83 (10)	C6—C7—H7	119.7
O2—S—C6	109.54 (10)	C9—C8—C7	120.8 (2)
N2—S—C6	106.98 (10)	C9—C8—H8	119.6
C3—N1—C4	122.20 (18)	C7—C8—H8	119.6
C3—N1—H1N	118 (2)	C8—C9—C10	119.9 (2)
C4—N1—H1N	120 (2)	C8—C9—H9	120.0
C1—N2—S	126.70 (16)	C10—C9—H9	120.0
C1—N2—H2N	122 (2)	C11—C10—C9	119.0 (2)
S—N2—H2N	110 (2)	C11—C10—H10	120.5
O4—N3—O3	123.2 (2)	C9—C10—H10	120.5
O4—N3—C11	117.7 (2)	C10—C11—C6	122.2 (2)
O3—N3—C11	119.09 (19)	C10—C11—N3	115.0 (2)
N2—C1—C2	122.01 (19)	C6—C11—N3	122.8 (2)
N2—C1—C5	118.34 (18)	F3^i^—Si—F3	89.03 (10)
C2—C1—C5	119.64 (18)	F3^i^—Si—F2	178.59 (7)
C3—C2—C1	118.97 (19)	F3—Si—F2	90.59 (7)
C3—C2—H2	120.5	F3^i^—Si—F2^i^	90.59 (7)
C1—C2—H2	120.5	F3—Si—F2^i^	178.59 (7)
N1—C3—C2	120.30 (19)	F2—Si—F2^i^	89.82 (9)
N1—C3—H3	119.8	F3^i^—Si—F1^i^	90.21 (7)
C2—C3—H3	119.8	F3—Si—F1^i^	92.20 (7)
N1—C4—C5	119.82 (19)	F2—Si—F1^i^	88.45 (7)
N1—C4—H4	120.1	F2^i^—Si—F1^i^	89.16 (7)
C5—C4—H4	120.1	F3^i^—Si—F1	92.20 (7)
C4—C5—C1	119.02 (19)	F3—Si—F1	90.21 (7)
C4—C5—H5	120.5	F2—Si—F1	89.16 (7)
C1—C5—H5	120.5	F2^i^—Si—F1	88.45 (7)
C11—C6—C7	117.3 (2)	F1^i^—Si—F1	176.62 (9)
C11—C6—S	125.73 (17)		
			
O1—S—N2—C1	−65.8 (2)	O2—S—C6—C7	131.78 (16)
O2—S—N2—C1	164.31 (18)	N2—S—C6—C7	−115.54 (17)
C6—S—N2—C1	48.2 (2)	C11—C6—C7—C8	−1.3 (3)
S—N2—C1—C2	24.9 (3)	S—C6—C7—C8	−177.48 (17)
S—N2—C1—C5	−155.30 (16)	C6—C7—C8—C9	0.6 (3)
N2—C1—C2—C3	−178.80 (19)	C7—C8—C9—C10	0.4 (3)
C5—C1—C2—C3	1.4 (3)	C8—C9—C10—C11	−0.6 (3)
C4—N1—C3—C2	−1.5 (3)	C9—C10—C11—C6	−0.1 (3)
C1—C2—C3—N1	0.3 (3)	C9—C10—C11—N3	−178.4 (2)
C3—N1—C4—C5	0.9 (3)	C7—C6—C11—C10	1.0 (3)
N1—C4—C5—C1	0.7 (3)	S—C6—C11—C10	176.86 (17)
N2—C1—C5—C4	178.30 (18)	C7—C6—C11—N3	179.2 (2)
C2—C1—C5—C4	−1.9 (3)	S—C6—C11—N3	−5.0 (3)
O1—S—C6—C11	−175.15 (18)	O4—N3—C11—C10	−43.6 (3)
O2—S—C6—C11	−44.1 (2)	O3—N3—C11—C10	132.9 (2)
N2—S—C6—C11	68.6 (2)	O4—N3—C11—C6	138.1 (3)
O1—S—C6—C7	0.70 (18)	O3—N3—C11—C6	−45.4 (3)

Symmetry codes: (i) −*x*+2, *y*, −*z*+3/2.

### Hydrogen-bond geometry (Å, °)

**Table:**

*D*—H···*A*	*D*—H	H···*A*	*D*···*A*	*D*—H···*A*
N1—H1N···F1^ii^	0.86 (3)	1.96 (3)	2.789 (2)	162 (3)
N2—H2N···F1^iii^	0.73 (3)	1.97 (3)	2.690 (2)	171 (3)
C4—H4···F2^ii^	0.95	2.35	3.141 (2)	141
C5—H5···F3^iii^	0.95	2.50	3.426 (3)	165

Symmetry codes: (ii) −*x*+3/2, *y*+1/2, −*z*+3/2; (iii) −*x*+3/2, *y*−1/2, −*z*+3/2.
